# Mesenchymal stem cells derived from iPSCs expressing interleukin-24 inhibit the growth of melanoma in the tumor-bearing mouse model

**DOI:** 10.1186/s12935-020-1112-7

**Published:** 2020-01-30

**Authors:** Zheng Wu, Wei Liu, Zujia Wang, Baitao Zeng, Guangnan Peng, Hongyan Niu, Linlin Chen, Cong Liu, Qian Hu, Yuxuan Zhang, Mengmeng Pan, Lingqian Wu, Mujun Liu, Xionghao Liu, Desheng Liang

**Affiliations:** 1 Center for Medical Genetics & Hunan Key Laboratory of Medical Genetics, School of Life Sciences, Central South University, Changsha, Hunan China; 2 Department of Cell Biology, School of Life Sciences, Central South University, Changsha, Hunan China; 3 Hunan Key Laboratory of Animal Model for Human Diseases, Central South University, Changsha, Hunan China

**Keywords:** iPSCs-derived MSCs, Human rDNA locus, Site-specific Integration, Interleukin-24, Melanoma

## Abstract

**Background:**

Interleukin-24 (*IL*-*24*) is a therapeutic gene for melanoma, which can induce melanoma cell apoptosis. Mesenchymal stem cells (MSCs) show promise as a carrier to delivery anti-cancer factors to tumor tissues. Induced pluripotent stem cells (iPSCs) are an alternative source of mesenchymal stem cells (MSCs). We previously developed a novel non-viral gene targeting vector to target *IL*-*24* to human iPSCs. This study aims to investigate whether MSCs derived from the iPSCs with the site-specific integration of *IL*-*24* can inhibit the growth of melanoma in a tumor-bearing mouse model via retro-orbital injection.

**Methods:**

IL-24-iPSCs were differentiated into IL-24-iMSCs in vitro, of which cellular properties and potential of differentiation were characterized. The expression of IL-24 in the IL-24-iMSCs was measured by qRT-PCR, Western Blotting, and ELISA analysis. IL-24-iMSCs were transplanted into the melanoma-bearing mice by retro-orbital intravenous injection. The inhibitory effect of IL-24-iMSCs on the melanoma cells was investigated in a co-culture system and tumor-bearing mice. The molecular mechanisms underlying IL-24-iMSCs in exerting anti-tumor effect were also explored.

**Results:**

iPSCs-derived iMSCs have the typical profile of cell surface markers of MSCs and have the ability to differentiate into osteoblasts, adipocytes, and chondroblasts. The expression level of IL-24 in IL-24-iMSCs reached 95.39 ng/10^6^ cells/24 h, which is significantly higher than that in iMSCs, inducing melanoma cells apoptosis more effectively in vitro compared with iMSCs. IL-24-iMSCs exerted a significant inhibitory effect on the growth of melanoma in subcutaneous mouse models, in which the migration of IL-24-iMSCs to tumor tissue was confirmed. Additionally, increased expression of Bax and Cleaved caspase-3 and down-regulation of Bcl-2 were observed in the mice treated with IL-24-iMSCs.

**Conclusion:**

MSCs derived from iPSCs with the integration of *IL*-*24* at rDNA locus can inhibit the growth of melanoma in tumor-bearing mouse models when administrated via retro-orbital injection.

## Background

Melanoma, a highly malignant skin tumor, is the main reason for death in skin cancer, 287,723 new melanoma cases occurred globally in 2018 [1]. The treatment is usually surgical resection. For patients with melanoma metastasis, treatment of immunotherapy [2–4], biotherapy [5], radiation therapy [6] or chemotherapy [7–13] is used to improve patient survival.

Interleukin-24/Melanoma differentiation-related gene-7 (*MDA*-*7/IL*-*24*) is a new member of the IL-10-associated cytokine gene family [14], and the *IL*-*24* gene encodes a 206-amino acid precursor protein that contains a 48-amino acid signal sequence and a mature fragment of 158 amino acids. IL-24 has been shown to induce apoptosis through intracellular and extracellular signaling mechanisms [15, 16].

MSCs are multipotential stromal cells that present in many tissues, such as bone marrow, umbilical cord and adipose tissue [17–19]. Several clinical trials have utilized MSCs to carry therapeutic factor, such as TK, TRAIL, to treat cancer [20, 21]. MSCs have been shown to be a promising delivery vehicle for cancer therapy owing to their ability to migrate to damaged tissue as well as tumor [22]. However, long culture period in vitro for MSCs expansion may increase the risk of chromosomal aberrations, in addition, limited proliferation potential in vitro could hamper their application because a large number of therapeutic MSCs is required for clinical trial. Recent studies showed iPSCs could be an alternative source of MSCs [23, 24]. Therefore, an unlimited number of MSCs expressing therapeutic gene could be generated through differentiation of genetically modified iPSCs. In this context, we have explored a strategy to modify iPSCs. Though majority of gene delivery in gene therapy is conducted by viral vectors, for safety concern, non-viral vectors are still of great interest. To achieve long-term expression of the transgene by a non-viral vector, We constructed a non-viral gene targeting vector, by which we targeted *IL*-*24* expression cassette into the ribosomal DNA locus of human iPSCs [25]. Our previous data showed that MSCs derived from human iPSCs with the integration of *IL*-*24* (IL-24-iPSCs) significantly inhibited the growth of melanoma cell when co-implanted into mice. In the present study, we differentiated IL-24-iPSCs to IL-24-iMSCs and investigated the anti-melanoma effect of IL-24-iMSCs on established tumor after retro-orbital injection into a tumor-bearing mouse model.

## Materials and methods

### Cell culture

The murine melanoma cells B16-F10 were purchased from ATCC and cultured in DMEM/HG (HyClone, USA) supplemented with 10% FBS (Gibco, USA). Human induced pluripotent stem cells (DYR0100) were purchased from ATCC and cultured in mTeSR1 medium (STEMCELL Technologies, Canada). IL-24-iPSCs was previously generated by our group. The MSCs derived from iPSCs were cultured in MSC medium with DMEM/LG (HyClone, USA) supplemented with 10% FBS and 0.1% bFGF (Sigma, USA). All cells were cultured at 37 °C in a humidified chamber maintained at 5% CO_2_.

### The differentiation of iPSCs into iMSCs

We used STEMdiff™ Mesenchymal Progenitor Kit (STEMCELL, USA) to differentiate iPSCs and IL-24-iPSCs into iMSCs and IL-24-iMSCs, respectively, according to the manufacturer’s protocol. Briefly, after iPSCs were cultured with mTeSR1 medium to a confluence of 30%, they were cultured with Mesenchymal Induction Medium for 4 days, and the medium was changed daily, and then cultured with MesenCult™-ACF Medium for 3 days. When the cell confluence reached 90%, they were passaged into a 6-well plate pre-coated with the MesenCult™-ACF attachment substrate, and the ACF medium was changed every day. After 4 days of cultured, cells with 90% confluency were passaged into a gelatin-coated 10-cm dish and continue to culture with MSC medium.

### Characterization of iMSCs and IL-24-iMSCs

The cell suspension was prepared at a concentration of 1 × 10^5^/mL in 1 × DPBS. 5 × 10^4^ cells were incubated with BV421-conjugated anti-human CD34, CD45 and HLA-DR, BB515-conjugated CD44,Precp-Cy5.5-conjugated CD73, APC-conjugated CD105 and PE-Cy7-conjugated anti-human CD90 (BD Biosciences, USA) at room temperature for 30 min. Stained cells were then washed twice in PBS. Flow cytometric analysis was performed by flow cytometer (BD Biosciences, USA) to detect the expression of cell surface markers of iMSCs and IL-24-iMSCs.

### Identification of differentiation potential of iMSCs

The differentiation potential of iMSCs was identified by Osteogenesis, Adipogenesis and Chondrogenesis Differentiation Kit (STEMPRO, Gibco). Briefly, cells were seeded in gelatin-coated 6-well plates at a concentration of 1 × 10^4^ cells/cm^2^, and cultured in MSC medium for 24 h at 37 °C in 5% CO_2_ saturated humidity incubator. 2 mL differentiation medium was then added to each well for differentiation culture. Fresh differentiation medium was changed every 3 days. After differentiation culture for 1 to 2 weeks, the cells were stained with an appropriate amount of Alizarin Red, Oil Red O and Alison Blue Dye for 30 min. After incubation, cells were washed with DPBS 3 times and dry, and were then analyzed by light microscopy.

### qRT-PCR

Total RNA was extracted using TRIzol reagent (Sigma-Aldrich, USA) and treated with DNase I (Thermo Fisher Scientific, USA) to eliminate genomic and other DNA. 50 ng RNA sample was reverse transcribed using HiScript^®^ II Q RT SuperMix (Vazyme, China). The q-PCR was performed on Bio-Rad CFX96 touch qPCR system (Bio-Rad, USA). The data analysis was performed using the Bio-Rad CFX Manager software (Bio-Rad, USA). Primers were designed to amplify exons 6 and 7 of the *IL*-*24* gene as follows: qPCR-IL-24-F: CAGGCGGTTTCTGCTATTC; qPCR-IL-24-R: GAATTTCTGCATCCAGGTCA). GAPDH was used as an internal control as follows: qPCR-GAPDH-F: AATCCCATCACCATCTTCCA; qPCR-GAPDH-R: TGGACTCCACGACGTACTCA).

### ELISA

After cultured iMSCs and IL-24-iMSCs in MSC medium for 3 days, 24 h-old supernatants were collected from 6-well plates. Total cells were digested and counted. All supernatants were collected in triplicate. ELISA was performed using Human Interleukin 24 (IL-24) ELISA Kit (Catalog# CSB-E15840h, CUSABIO) according to the manufacturer’s instructions.

### Co-culture experiments

iMSCs or IL-24-iMSCs (3 × 10^4^ cells) were seeded in upper well of 6-well transwell plate (0.4 μm PET MEM, Corning, USA). B16-F10 cells (1 × 10^4^/well) were seeded in lower well. The transwell plates were cultured using MSC medium for 7 days. After 7 days, B16-F10 cells were harvested for apoptosis assay and western blotting assay.

B16-F10 cells were harvested and stained with AnnexinV-FITC/PI Apoptosis Detection Kit (Vazyme, China) according to the manufacturer’s instructions, followed by flow cytometry and analysis to estimate the percentage of apoptotic cells (AnnexinV positive).

### Apoptosis detection

Three groups, B16-F10 cells alone, B16-F10 cells co-cultured with iMSCs, B16-F10 cells co-cultured with IL-24-iMSCs, were set up. Cells were digested with TrypLE Select (Gibco, USA), 5 × 10^4^ cells were collected and incubated with Annexin V FITC Conjugate and propidium iodide solution from Annexin V-FITC Apoptosis Detection Kit (Vazyme, China) for 10 min at room temperature in the dark, followed by analysis using flow cytometry (BD Biosciences, USA).

### Western blotting

Cell lysates from B16-F10 cells, tumor tissues and MSCs were prepared by RIPA Lysis Buffer (Beyotime, China) and quantified by Pierce™ BCA Protein Assay Kit (Thermo, USA). Twenty micrograms of protein from each sample were was loaded for electrophoresis by SDS-PAGE and transferred to PVDF membranes (Merck KGaA, Germany). After blocking, the membranes were incubated with primary and secondary antibodies sequentially. The protein band was detected with an enhanced chemiluminescence kit (SuperSignal™ West Femto Maximum Sensitivity Substrate, Thermo). Anti-mouse Cleaved caspase-3 (1:1000) were obtained from Cell Signaling Technology (Danvers, MA). Anti-mouse Cleaved PARP (1:1000) were obtained from Santa Cruz Biotechnology (Santa Cruz, CA). Anti-mouse Bcl-2 (1:1000) was obtained from Santa Cruz Biotechnology (Santa Cruz, CA). Anti-mouse Bax (1:1000) was obtained from Genetex (Irvine, CA). Anti-human IL-24 (1:1000) was obtained from R&D Systems (USA).

### Animal studies

Four-week-old male C57BL/6 mice were purchased from the Laboratory Animal Center of Shanghai Academy of Sciences (Shanghai, China). The animal studies were approved by the Ethics Committee for Animal Experimentation of Central South University in China (NO:201601-20). IL-24-iMSCs and iMSCs were labeled with 5 μM CM-Dil (Invitrogen, USA) according to the manufacturer’s instructions. For the generation of melanoma mouse models, 9 mice were equally divided into three groups, 5 × 10^5^ of B16-F10 cells were subcutaneously injected into the inguinal region of each mouse. One week later, 1 × 10^6^ of CM-Dil labeled iMSCs or CM-Dil labeled IL-24-iMSCs were implanted into the tumor-bearing mice by retro-orbital intravenous injection, which were given two times with a week interval. Tumor sizes were measured by vernier calipers every 2 days, and the volumes were calculated with the formula: volume = (width^2^ × length) × π/6. Anesthetized comatose mice were sacrificed by cervical dislocation on the 19th day after xenograft. Tumor tissues were collected, photographed, weighed.

### Immunofluorescence and histological analysis

Tumor tissues were harvested, half of which were used for frozen sections (10 μm thickness), another half of which were fixed with 4% paraformaldehyde for paraffin sections (4 μm thickness). For detection of IL-24, sections were incubated with anti-human IL-24 antibody (dilution of 1:100, rabbit polyclonal, Catalog# ab115207, Abcam, USA), followed by incubation with fluorescent AF488 anti-rabbit secondary antibody (dilution of 1:100, Catalog# 111-035-244, Jackson ImmunoResearch). CM-Dil and fluorescence staining was visualized and acquired by the confocal fluorescent microscopy.

Paraffin sections (4 μm thickness) were stained with hematoxylin and eosin (H&E) for histological examination.

### Statistical analysis

The Student’s t-test and one-way ANOVA were used for data analysis of different experimental groups by GraphPad Software. Data were expressed as the mean ± standard error of the mean. p < 0.05 was considered statistically significant.

## Result

### Differentiation of iPSCs into iMSCs

**Fig. 1:**
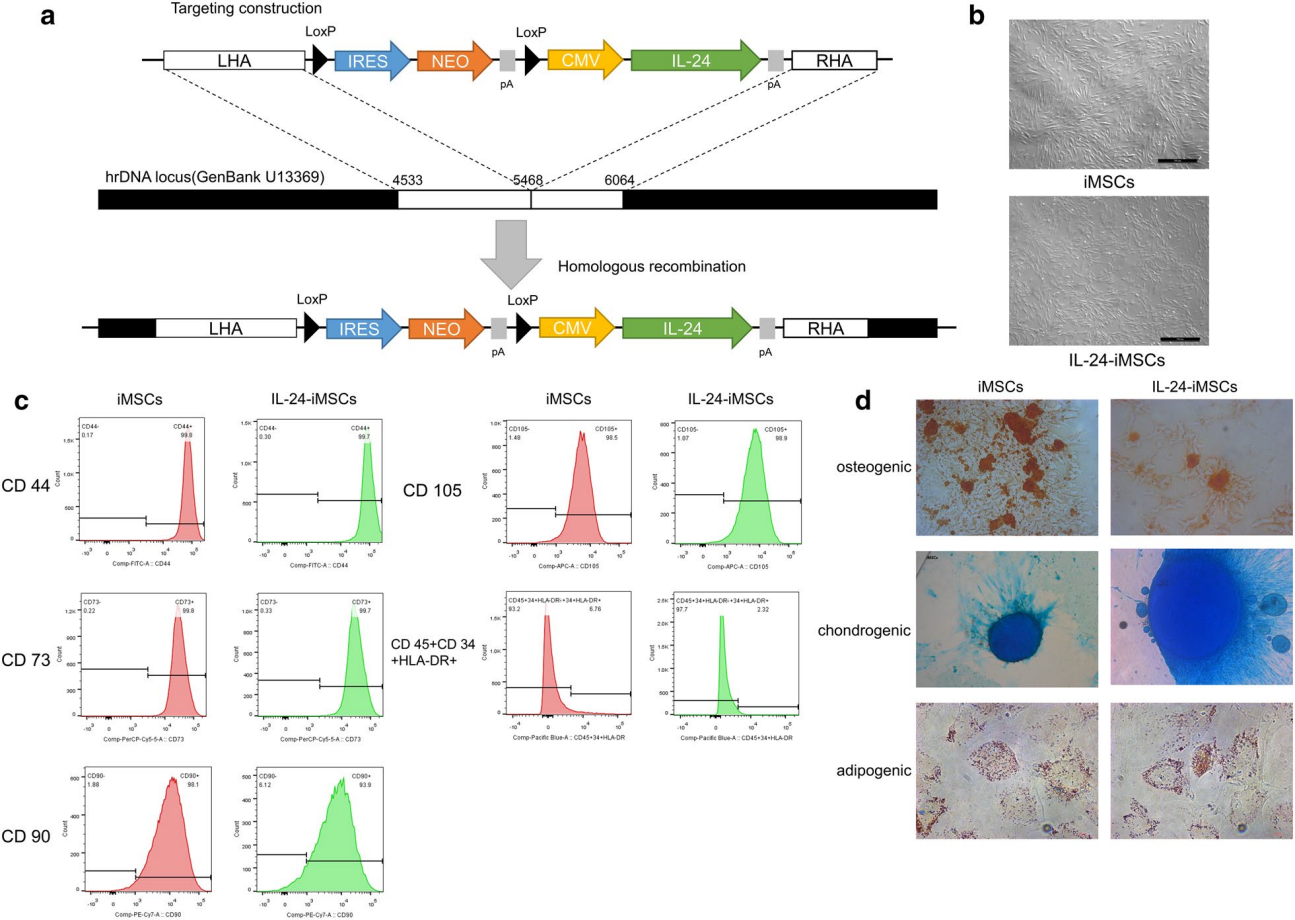
Generation and characterization of iMSCs. **a** Schematic representation of the gene targeting vector is shown. The left homologous arm (LHA) and right homologous arm (RHA) are shown by white boxes. The neomycin cassette consists of an IRES element, the coding region of the neomycin gene (NEO), and SV40 polyA signal (SpA). The *IL*-*24* gene is driven by a CMV promoter. **b** Morphology of the MSCs derived from iPSCs and IL-24-iPSCs, named as iMSCs and IL-24-iMSCs. **c** Flow cytometric analysis of surface markers in iMSCs and IL-24-iMSCs (n = 3 for each group). **d** Analysis of adipogenic, osteogenic, and chondrogenic differential potential of IL-24-iMSCs and iMSCs

In the previous study, we developed a novel non-viral rDNA region targeting vector, minipHrn-IL-24, by which *IL*-*24* was targeted into the ribosomal DNA locus of hiPSCs (Fig. 1a) In this study, we differentiated iPSCs with and without the integration of *IL*-*24* into MSCs, named IL-24-iMSCs and iMSCs, respectively. Our results revealed that IL-24-iMSCs and iMSCs showed a typical fibroblast-like morphology (Fig. 1b). Detection of cell surface marker by flow cytometry indicated that IL-24-iMSCs and iMSCs were negative for CD34, CD45, HLA-DR and positive for CD44, CD73, CD90, and CD105, which is similar to human MSCs (Fig. 1c). To assess the multi-lineage potential of IL-24-iMSCs and iMSCs, we performed osteogenic, adipogenic, and chondrogenic differentiation assay. For osteogenic differentiation, IL-24-iMSCs and iMSCs were positively stained with alizarin red S. For adipogenic differentiation, IL-24-iMSCs and iMSCs were positively stained with oil red O. For chondrogenic differentiation, IL-24-iMSCs and iMSCs were positively stained with alcian blue. These results demonstrated that IL-24-iMSCs and iMSCs displayed typical characters of MSCs.

### IL-24-iMSCs express exogenous IL-24

**Fig. 2:**
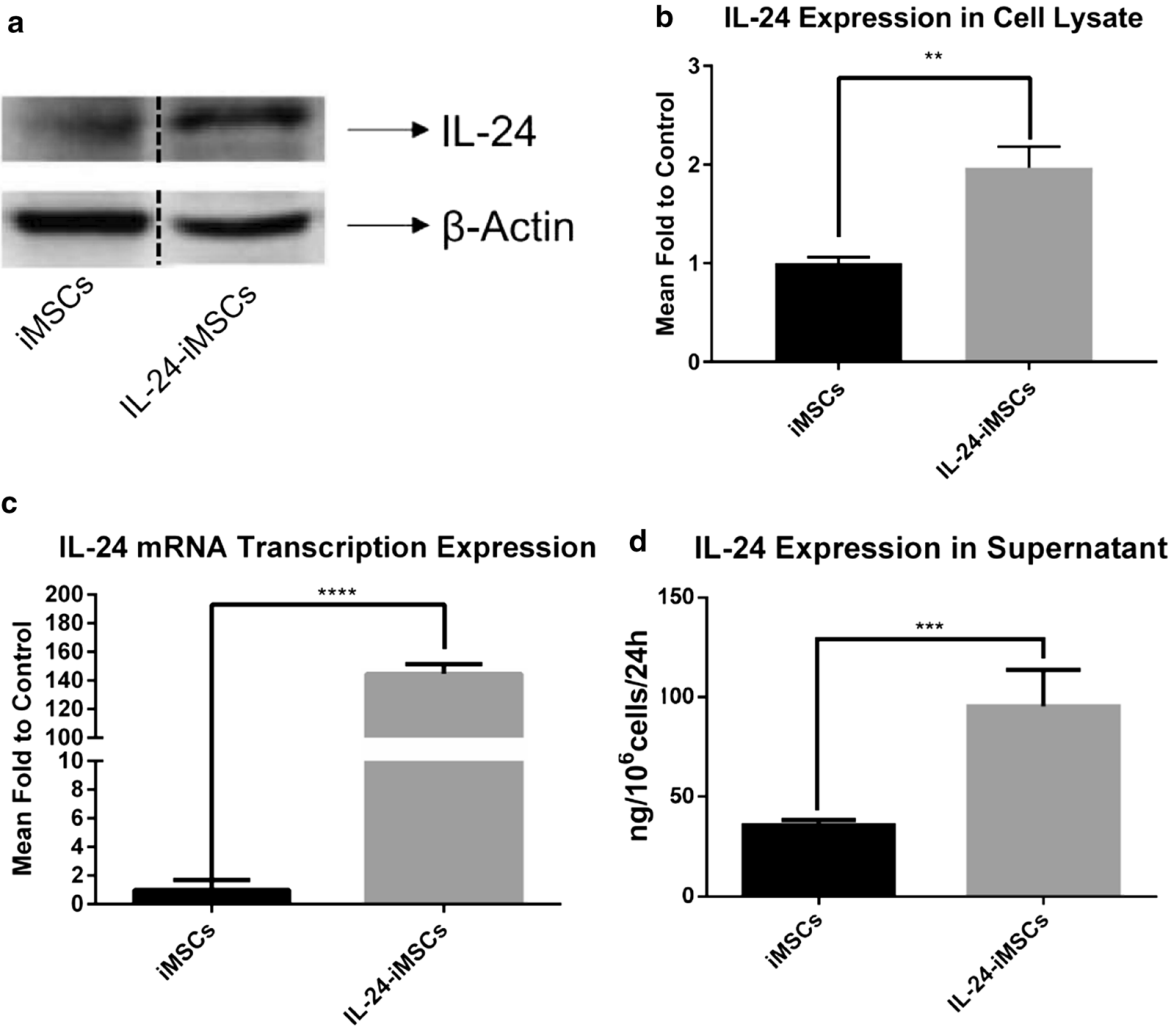
Interleukin-24 expression assay. **a**, **b** Western blot analysis of cell lysates from iMSCs and IL-24-iMSCs. The expression levels of IL-24 in IL-24-iMSCs were about twofold higher than in control iMSCs (data are mean ± SEM; n = 3 for each group; ***p < 0.001, ****p < 0.0001, One way ANOVA). **c** IL-24 mRNA transcript levels were detected by qRT-PCR in iMSCs and IL-24-iMSCs. The transcription level of IL-24 in IL-24-iMSCs was much higher than that of iMSCs (n = 3 per group; **p < 0.01, ***p < 0.001, ****p < 0.0001, One-way ANOVA). **d** ELISA analysis of IL-24 in the supernatants from iMSCs and IL-24-iMSCs is shown. IL-24 levels in the supernatants of iMSCs and IL-24-iMSCs were 37.55 ng and 95.39 ng, respectively in 10^6^ cells in 24 h (data are mean ± SEM; n = 3 for each group; ****p < 0.0001 by One-way ANOVA)

The level of IL-24 protein expression in cell lysates of IL-24-iMSCs was twofold higher than that of iMSCs (Fig. 2a, b). The result of qRT-PCR showed that IL-24 mRNA level in IL-24-iMSCs is much higher than that in iMSCs, approximately 140-fold (Fig. 2c). The IL-24 level in the culture supernatants of IL-24-iMSCs and iMSCs were 37.55 ng/10^6^ cells/24 h and 95.39 ng/10^6^ cells/24 h, respectively (Fig. 2d).

### IL-24-iMSCs induce apoptosis of melanoma cells in vitro

**Fig. 3:**
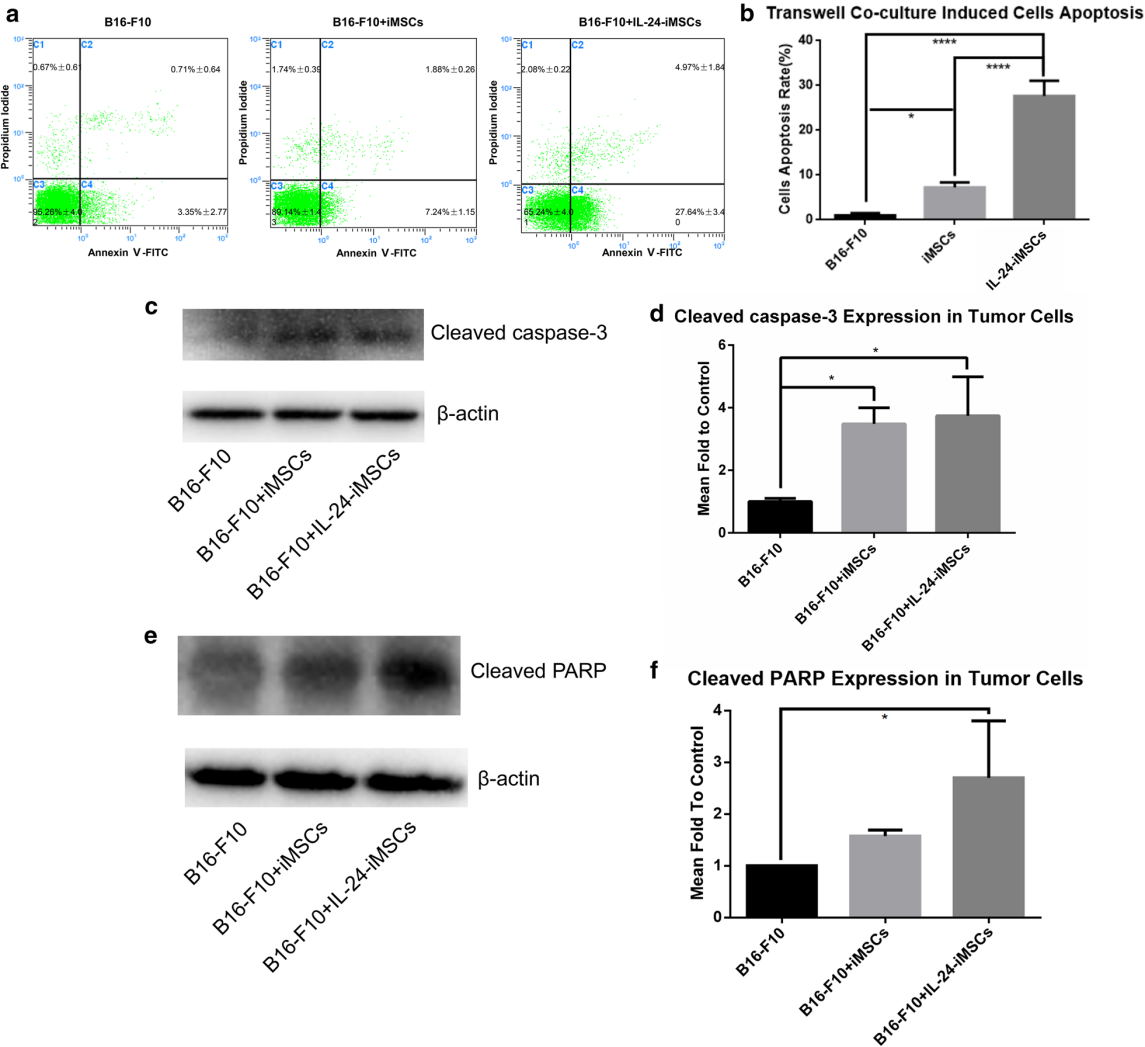
IL-24-iMSCs induce melanoma cell apoptosis in vitro. **a**, **b** Flow cytometric analysis of cell apoptosis assay. B16-F10 cells co-cultured with IL-24-iMSCs showed increased apoptosis compared to co-culturing with control iMSCs or B16-F10 cells culture alone in vitro (data are mean ± SEM; n = 3 for each group; *p < 0.05, **p < 0.01, ***p < 0.001, ****p < 0.0001, One way ANOVA). **c**, **d** The expression of Cleaved caspase-3 were examined in B16-F10 cells culture alone (control group) and co-culture with IL-24-iMSCs, iMSCs by Western blotting analysis (data are mean ± SEM; n = 3 for each group; *p < 0.05, **p < 0.01, ***p < 0.001, ****p < 0.0001, One way ANOVA). **e**, **f** The expression of Cleaved PARP were examined in B16-F10 cells culture alone (control group) and co-culture with IL-24-iMSCs, iMSCs by Western blotting analysis (data are mean ± SEM; n = 3 for each group; *p < 0.05, **p < 0.01, ***p < 0.001, ****p < 0.0001, One way ANOVA)

To investigate whether IL-24-iMSCs can induce apoptosis in melanoma cells in vitro, we co-cultured mouse melanoma cells (B16-F10) with IL-24-iMSCs, or with iMSCs, followed by incubated with Annexin V-FITC and PI antibody. Analysis of flow cytometry showed that the apoptosis rate of B16-F10 cells co-cultured with IL-24-iMSCs was 28%, whereas the apoptosis rate of B16-F10 cells co-cultured with iMSCs was 7% (Fig. 3a, b). in addition, we detected higher expression of cleaved PARP and cleaved Caspase-3 in group of B16-F10 cells co-cultured with IL-24-iMSCs compared to either group of B16-F10 cells alone or B16-F10 cells co-cultured with iMSCs (Fig. 3c, d). These results show that both IL-24-iMSCs and iMSCs can induce melanoma cell apoptosis in vitro, and that IL-24-iMSCs are more effective than iMSCs.

### IL-24-iMSCs inhibit the growth of melanoma cells in tumor-bearing mice by intravenous implantation

**Fig. 4:**
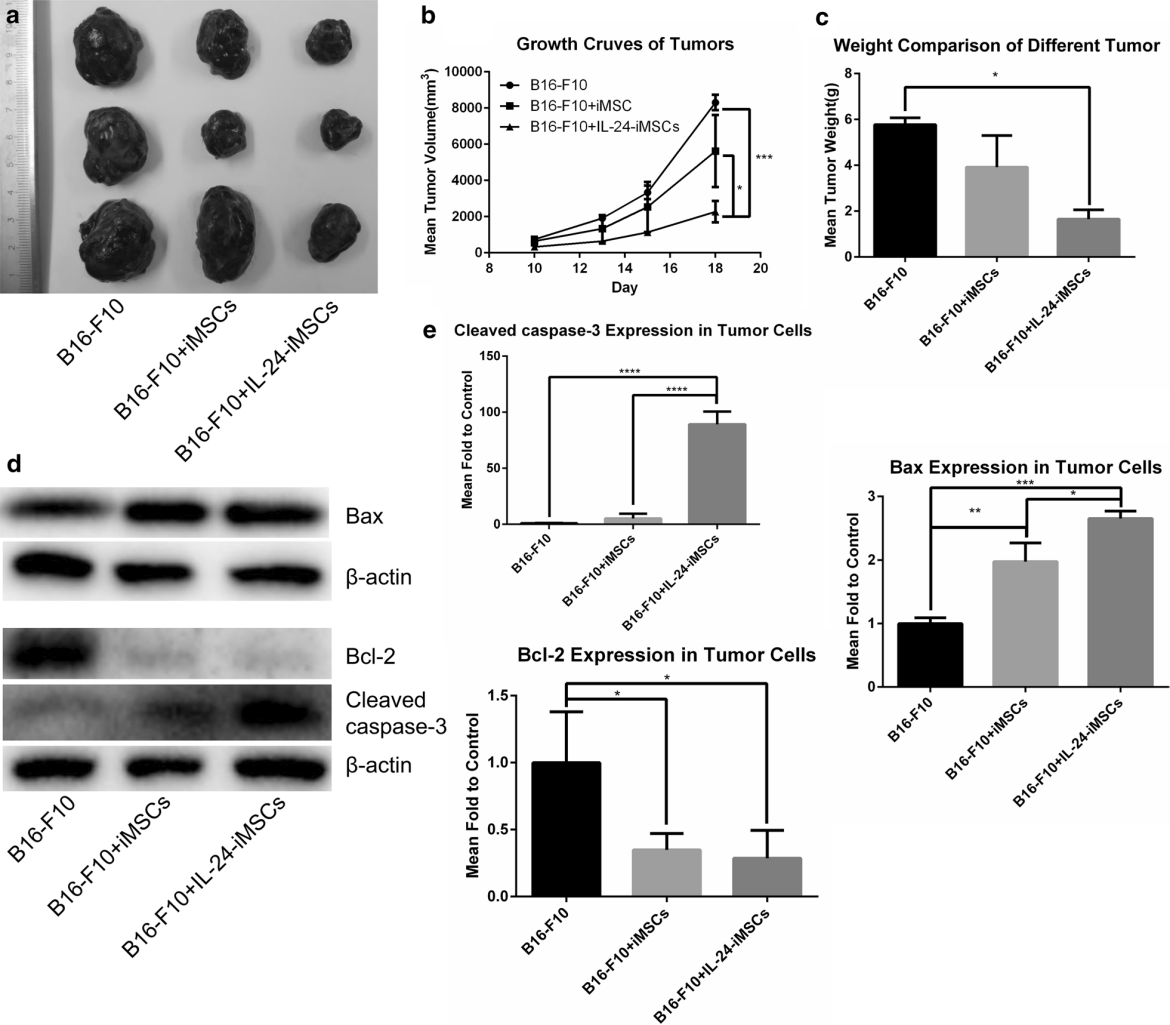
Inhibition of tumor growth by IL-24-iMSCs in vivo. **a** Morphology of tumor tissues in the control group (B16-F10 alone), the group treated with iMSCs, and the group treated with IL-24-iMSCs. **b** The process of tumor size over time in mice on the period of 18 days. The average tumor volume on day 18 was 8303.52 mm^3^ in the control group, and the average tumor volume on the 18th day was 5620.24 mm^3^ in the group treated with iMSCs. The mean tumor volume of the group treated with IL-24-iMSCs was 2067.74 mm^3^ on day 18 (n = 3 per group; *p < 0.05, **p < 0.01, ** *p < 0.001, ****p < 0.0001, One-way ANOVA). **c** Measurement of tumor weights from C57BL/6 mice injected with or without IL-24-iMSCs at the time of sacrifice. Tumor weight was significantly reduced the group treated with IL-24-iMSCs (n = 3 per group; *p < 0.05, **p < 0.01, ***p < 0.001, ****p < 0.0001, One-way ANOVA). **d**, **e** The expression levels of proteins related to apoptosis were examined in tumors injected with IL-24-iMSCs, iMSCs and untreated (control group) by Western blotting analysis (data are mean ± SEM; n = 3 for each group; *p < 0.05, **p < 0.01, ***p < 0.001, ****p < 0.0001, One way ANOVA)

**Fig. 5:**
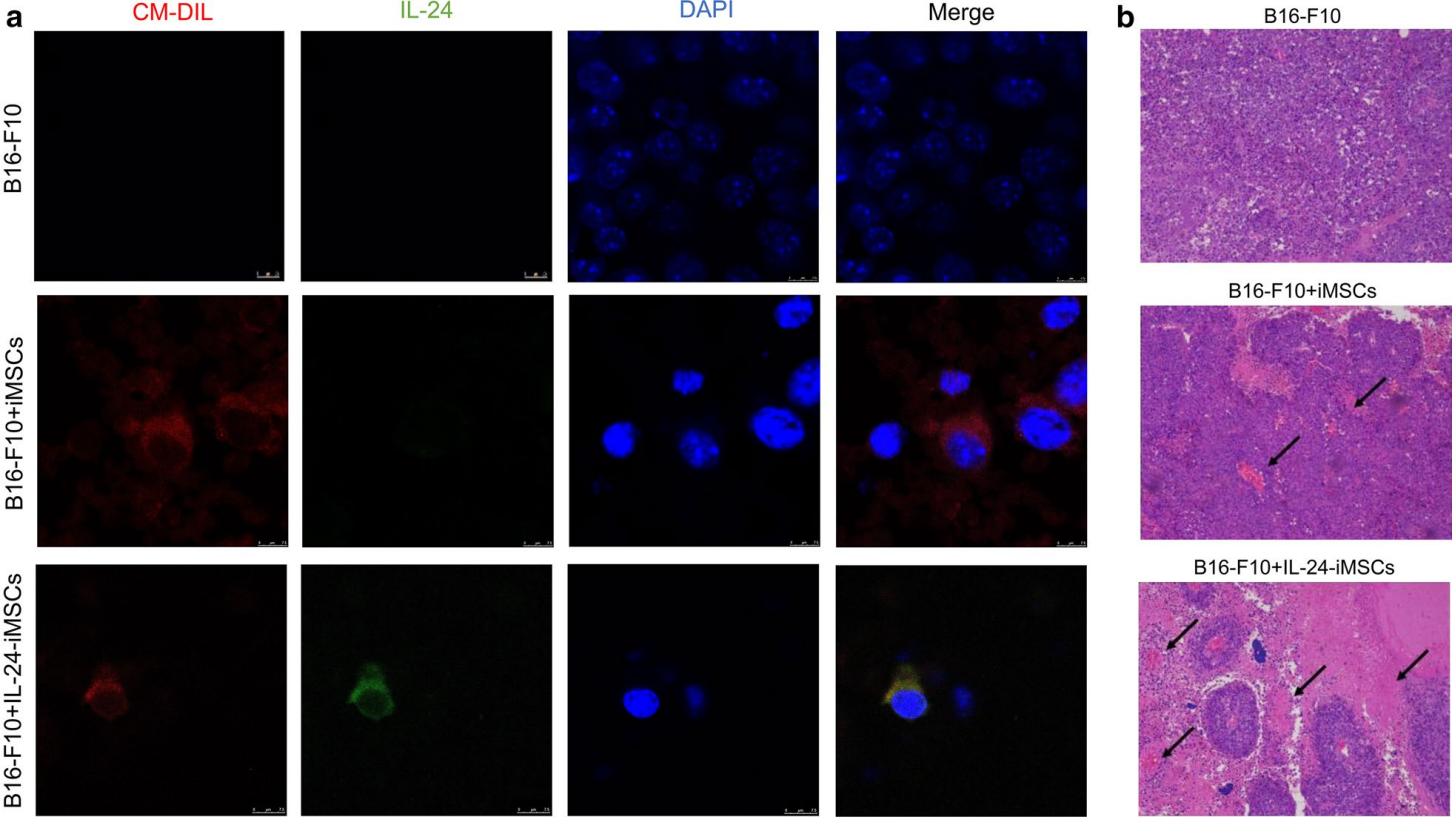
Both iMSCs and IL-24-iMSCs migrate to tumor tissues (**a**). Immunofluorescence analysis was performed to detect the expression of human IL-24 in the control group (B16-F10 alone), the group treated with iMSCs, and the group treated with IL-24-iMSCs. Melanoma tissue section assay showed CM-Dil (red fluorescence), human IL-24 protein (green fluorescence) and nuclear DAPI staining (blue) (**b**). H&E stained histological sections of tumors showed that there were necrotic areas (arrows) in the group treated with IL-24-iMSCs and the group treated with iMSCs

To assess the anti-cancer effect of IL-24-iMSCs in the tumor-bearing mouse model, we performed subcutaneous injection of 5 × 10^5^ B16-F10 cells in the C57BL/6 to generate melanoma-bearing mice. We used CM-Dil to label IL-24-iMSCs and iMSCs before administration. CM-Dil labeled iMSCs and IL-24-iMSCs were transplanted into the mouse models through retro-orbital injection. The growth curve of melanoma revealed that both IL-24-iMSCs and iMSCs inhibited the growth of B16-F10. Group of control (B16-F10 alone) formed tumors with an average tumor volume of 8303.52 mm^3^ on day 18. Group of iMSCs formed tumors with an average tumor volume of 5620.24 mm^3^ on day 18. Group of IL-24-iMSCs formed tumors with an average tumor volume of 2067.74 mm^3^ on day 18 (Fig. 4a, b). The average weight of tumor tissue in control was 3.5-fold heavier than that of the IL-24-iMSCs group, 1.5-fold heavier than that of the iMSCs group (Fig. 4c). Histological analysis of melanoma tissue sections indicated that there were necrotic areas and nuclear agglomeration areas in the tumors in IL-24-iMSCs group and iMSCs group. In contrast, the tumor necrosis area in the tumor sections injected with IL-24-iMSCs group was more than that in the iMSCs group (Fig. 5b). Importantly, Immunofluorescence analysis demonstrates that both CM-Dil-labeled IL-24-iMSCs with the expression of IL-24 and iMSCs exist in melanoma of the treated group (Fig. 5a). In addition, to investigate whether B16-F10 underwent apoptosis in treated models, we tested the expression of pro-apoptotic proteins and pro-survival proteins in tumor tissues. We observed an increased expression of pro-apoptotic protein Bax and Cleaved caspase-3 but a reduced expression of the pro-survival protein Bcl-2 in IL-24-iMSCs group (Fig. 4d, e).

## Discussion

Currently, many studies have demonstrated the ability of MSCs to migrate to the tumor in different preclinical cancer models including colorectal cancer [26], Kaposi sarcoma [27], breast cancer [28] and lung tumor [29]. In this context, MSCs can be used as a carrier to delivery anti-cancer factor for tumor-targeted therapies. MSCs with the expression of different cytokines, such as IFN-β, IFN-α, IL-12, exerted an anti-cancer effect in a variety of cancer models including pancreatic tumors [30], lung metastasis of melanoma [31], renal cell carcinoma [32]. The molecular mechanisms of tumor tropism of MSCs may be related to various cytokine and their corresponding cytokine receptors. Tumors can release chemokines and cytokines by autocrine or paracrine, promoting MSCs with a large number of receptors to migrate to tumors [33]. In the present study, CM-DIL labeled iMSCs and IL-24-iMSCs were found in established melanoma of mouse models after retro-orbital injection, indicating that MSCs derived from iPSCs can also be recruited to the site of the tumor.

Although MSCs can be readily obtained from various tissues, such as bone marrow and umbilical cord, variations of both quantity and quality of MSCs from different sources could affect their clinical applications. Besides, the increase of expansion rates in vitro could limit their potential for proliferation and differentiation [34]. Current studies have shown that iPSCs is an alternative source of MSCs [23, 24]. In this context, iPSCs can be genetically modified to express anti-cancer factors, from which a large number of therapeutic MSCs with relative uniform quality can be derived. We have previously constructed a novel non-viral targeting vector, the ribosomal DNA targeting vector (pHrneo), which has targeted therapeutic genes into the rDNA locus of different human cell types including hepatocyte cell lines [35], MSCs [36], and embryonic stem cells [37]. More importantly, we have used this vector to target *IL*-*24* into the ribosomal DNA locus of human iPSCs and obtained an iPSC cell line with the expression of exogenous gene *IL*-*24* (IL-24-iPSCs). In the present study, we differentiated IL-24-iPSCs into MSCs (IL-24-iMSCs). Our results showed that IL-24-iMSCs express typical panel of MSC surface markers [CD105(+), CD73(+), CD90(+), CD44(+), CD34(−), CD45(−), HLA-DR(−)], and possess the ability to differentiated into osteoblasts, adipocytes, and chondroblasts. The IL-24 expression level of IL-24-iMSCs reached 95.39 ng/10^6^ cells/24 h, which was comparable to that of IL-24-iMSCs obtained from our previous studies, indicating that IL-24-iMSCs can stably express IL-24.

In this study, we injected iMSCs and IL-24-iMSCs into tumor-bearing mice by retro-orbital intravenous injection. We found that IL-24-iMSCs could inhibit the growth of melanoma in tumor-bearing mice more significantly, which was related to the anti-tumor effect of IL-24. IL-24 is a tumor suppressor [16, 38–40], which can promote apoptosis of tumor cells [16, 41], inhibit angiogenesis [42], stimulate immune response [43] and synergize with other drugs [44, 45] to enhance anti-tumor efficacy. IL-24 can induce cell death by activating PERK that in turn leads to decreased expression of pro-survival genes *BCL*-*2* and *BCL*-*XL* [46, 47] and increased expression of pro-apoptotic genes like *BAX* and *BAK* [48–50], eventually leading to the activation of Caspase-3 and Caspase-9 [51, 52]. Our results also confirmed up-regulation of Bax and caspase-3 and down-regulation of Bcl-2 in melanoma of mouse models after administration of IL-24-iMSCs, demonstrating that IL-24 contributed to the anti-tumor effects of IL-24-iMSCs.

## Conclusion

In conclusion, we differentiated the IL-24-iPSCs with the integration of *IL*-*24* at the ribosomal DNA locus into IL-24-iMSCs. Our results demonstrated that IL-24-iMSCs significantly inhibited the growth of melanoma in tumor-bearing mice after systemic administration. IL-24-iPSCs is thought to be promising for the development of off-the-shelf therapeutic MSCs in cancer therapy.

## Data Availability

The datasets generated and/or analyzed during the current study are available from the corresponding author on reasonable request.

## Abbreviations

bFGF: basic fibroblast growth factor
CD: cluster of differentiation
DAPI: 4,6-diamidino-2-phenylindole
DMEM: Dulbecco’s minimum essential medium
ELISA: enzyme-linked immunosorbent assay
FBS: fetal bovine serum
FITC: fluorescein isothiocyanate
GAPDH: glyceraldehyde-3-phosphate dehydrogenase
hiPSCs: human induced pluripotent stem cells
IL: interleukin
iMSC: induced mesenchymal stem cell
MDA-7: melanoma differentiation-association gene 7
PERK: protein kinase RNA-like endoplasmic reticulum kinase
PI: propidium iodide
rDNA: ribosomal DNA
